# Stress among nursing staff and interventions in Austrian nursing homes: Results of a survey in the first and the second waves of COVID-19

**DOI:** 10.1007/s16024-022-00395-x

**Published:** 2023-02-02

**Authors:** Silvia Bauer, Doris Eglseer, Manuela Hödl

**Affiliations:** Institute of Nursing Science, Medical University of Graz, Universitätsplatz 4/3, 8010 Graz, Austria

**Keywords:** Burden, First wave, Second wave, Associated factors, Long-term care, Belastung, Erste Welle, Zweite Welle, Einflussfaktoren, Langzeitpflege

## Abstract

**Background:**

Most of the limited number of studies that have been carried out on COVID-19 in nursing homes have not included primarily nursing staff. Nevertheless, knowledge about staff experiences will help to provide recommendations for the future.

**Aim:**

The aim of this study was to describe stress experienced and interventions performed by nursing staff and to identify factors that are associated to the perceived stress among Austrian nursing home staff during the first and the second waves of COVID-19.

**Methods:**

A secondary data analysis of two cross-sectional surveys performed in 2020 and 2021 among nursing home staff was performed. We did descriptive analysis as well as univariate and multivariate logistic regression analyses.

**Results:**

A total of 449 nurses participated in the first survey and 300 in the second survey. 12.7% experienced high stress levels in the first wave, while 26.0% experienced high stress levels in the second wave (*p* < 0.001). The analysis showed that nursing staff in the second wave had a 2.195-fold higher relative chance of experiencing a high stress level compared to nursing staff in the first wave (*p* < 0.001). Caring for COVID-19 residents (odds ratio [OR] 1.827; *p* = 0.007) and being female (OR 1.992; *p* = 0.018) also significantly increased the relative chance of experiencing a high stress level. Some protective interventions, such as the use of FFP masks, increased between the two waves, while others decreased, such as the practice of airing the residents’ rooms.

**Conclusion:**

Austrian nursing staff in nursing homes experienced more stress during the second wave, illustrating the heavy burden of the long pandemic on staff. Nursing management should plan appropriate supportive interventions such as psychological help, stress relief measures and financial incentives for nursing staff, especially for the identified high-risk groups.

**Supplementary Information:**

The online version of this article (10.1007/s16024-022-00395-x) contains supplementary material, which is available to authorized users.

## Abstract (de)

**Hintergrund:**

Es gibt nur wenige Studien zum Thema COVID-19 in Pflegeheimen, die sich primär mit Pflegepersonen beschäftigen. Für die Zukunft könnten jedoch Informationen über die Erfahrungen des Pflegepersonals bei der Erstellung von Empfehlungen hilfreich sein.

**Ziel:**

Ziel dieser Studie war es den wahrgenommenen Stress, die durchgeführten Interventionen bei Pflegepersonen und Faktoren, die mit dem Stress zusammenhängen, in österreichischen Pflegeheimen während der ersten und zweiten COVID-19-Welle zu beschreiben.

**Methoden:**

Es wurde eine Sekundärdatenanalyse von zwei Querschnittsstudien bei Pflegepersonen in den Jahren 2020 und 2021 gemacht. Wir führten eine deskriptive Analyse und eine univariate bzw. multivariate logistische Regression durch.

**Ergebnisse:**

Insgesamt nahmen 449 Pflegepersonen an der ersten Erhebung und 300 Pflegepersonen an der zweiten Erhebung teil. 12,7 % der Pflegepersonen hatten in der ersten Welle hohe Stresslevel während 26 % in der zweiten Welle hohe Stresslevel hatten (*p* < 0,001). Die Analyse zeigte, dass Pflegepersonen in der zweiten Welle eine 2,195-fach höhere relative Wahrscheinlichkeit hatten, hohe Stresslevel zu haben im Vergleich zu Pflegepersonen in der ersten Welle (*p* < 0,001). Frauen (OR 1,992; *p* = 0,018) und Pflegepersonen, die Personen mit COVID-19 betreuen (OR 1,827; *p* = 0,007) haben ebenfalls eine erhöhte relative Wahrscheinlichkeit für hohe Stresslevel. Einige Schutzmaßnahmen, wie beispielsweise die Nutzung von FFP2-Masken, stiegen zwischen der ersten und zweiten Welle an, während wiederum andere abnahmen, wie beispielsweise das Lüften der Räume der Bewohner*innen.

**Schlussfolgerung:**

Pflegepersonen in österreichischen Pflegeheimen erfuhren, während der zweiten COVID-19-Welle hohe Stresslevel, wodurch die hohe Belastung durch die lang andauernde Pandemie sichtbar wird. Das Pflegemanagement sollte adäquate Unterstützungsmaßnahmen, wie psychologische Hilfsmaßnahmen, Maßnahmen zur Stressreduzierung und finanzielle Hilfestellungen für das Pflegepersonal und vor allem für die identifizierten Hochrisikogruppen, planen.

## Introduction

Since March 2020, the COVID-19 pandemic has exerted control over the whole world (WHO 2020a). The main aims of all countries are more or less the same: to protect high-risk groups (Centers for Disease Control and Prevention 2021) and to prevent a collapse of the healthcare systems (WHO 2020b).

The frontline healthcare staff play a major role in ensuring how well the healthcare system function. Members of this frontline staff, including nurses are the most heavily involved in the care of suspected and affected COVID-19 cases (Galehdar et al. 2021). These staff are available at the bedside 24/7. They have the responsibility to care for the patients and residents and to apply preventive interventions to ensure that they do not infect someone or become infected by someone (BBC News 2020; Rimmer and Madden 2021).

The first wave of COVID-19 arrived in Europe and, therefore, also in Austria in spring of 2020. The high infection rates combined with unclear regulations and a lack of sufficient personal protective equipment resulted in a chaotic situation in healthcare institutions (Bauer et al. 2020). In the second wave that arrived in Austria in the autumn of 2020, the infection rates reported were higher than those reported in the first wave (AGES 2021; WHO 2021). In addition, more nurses and nursing aids were infected or were classified as a contact person 1; therefore, they had to enter quarantine. This occurred not only in Austria, but also in other countries (AGES 2021; McGilton et al. 2020) and led to staff shortages and high workloads (Ouslander and Grabowski 2020) and may have also led to much higher stress levels as compared to those experienced in the first COVID-19 wave.

Nursing homes are healthcare institutions that provide care for the most vulnerable members of the population and have been strongly affected by the pandemic (Ouslander and Grabowski 2020; McGilton et al. 2020). Internationally, the proportion of nursing home resident deaths as compared to all COVID-19 deaths range from 8% in Slovenia to 75% in Australia (Comas-Herrera et al. 2021). In Austria, 44% of all COVID-19-attributed deaths were nursing home residents (Comas-Herrera et al. 2021).

However, the nursing home staff members are at high risk of being infected with the COVID-19 virus as well. A recent report showed that the COVID-19 incidence among nursing home staff was as high as the incidence rates among nursing home residents from July to November 2020 (Bagchi et al. 2021). This high infection risk places a huge burden on the nursing home staff. Therefore, the WHO considered protecting the mental health of the nursing staff as one main aspect of occupational safety issues during the COVID-19 pandemic (WHO & International Labour Organization 2021). Nursing staff who experience uncertainty regarding whether they are infectious or not can be placed under high levels of emotional and psychological pressure. On the one hand, they are afraid of infecting the residents (BBC News, 2020), as these form one of the most vulnerable groups in this pandemic (McGilton et al. 2020). On the other hand, they are afraid of infecting their own families at home. This aspect has also been mentioned by nursing staff as one reason why some of them have lived in caravans during the lockdown (Rimmer and Madden 2021).

In addition, the need to work with personal protective equipment, staff shortages, longer working shifts, fewer breaks and other factors may influence the stress level of the nursing staff. Nevertheless, stress is also influenced by the need to carry out necessary interventions to protect themselves and to protect the residents they care for. With respect to the management of COVID-19 in the nursing home, these interventions included monitoring the residents’ symptoms, taking their temperature and airing the rooms twice a day (Federal Ministry for Social Affairs, Health, Care and Consumer Protection 2020a, b). The need to carry out such additional interventions inevitably makes managing COVID-19 in the nursing home more difficult and make an already challenging and stressful job even more so (Leskovic et al. 2020).

In a thorough literature review, we did not identify many studies that reported the stress levels perceived and interventions performed by nursing staff during the COVID-19 pandemic. We also found no studies on factors that are associated with such perceived stress. To our knowledge, only a limited number of studies have placed a focus on the nursing home setting, most of which did not include primarily nursing staff. This information, however, would provide valuable insights into and explanations for this perceived stress and may help to provide future recommendations as well as strategies that can be applied to prevent such high stress levels among nursing staff.

## Aim

The aim of this study was to describe the stress perceived, the interventions performed by nursing staff and to identify factors that are associated with the perceived stress among Austrian nursing home staff during the first and the second waves of the COVID-19 pandemic.

## Methods

### Design, setting and sample size

This study involved a secondary analysis of data collected with two cross-sectional online surveys. The first survey was performed during the first COVID-19 wave in Austria (spring 2020) in order to describe the quality of nursing care provided during the pandemic (Bauer et al. 2020; Hödl et al. 2021c). The second survey was conducted during the second wave of COVID-19 in Austria (autumn and winter 2020/2021) to achieve the same aim (Hödl et al. 2021b; Schoberer et al. 2022). In both cases, nursing staff from different healthcare organizations were invited to participate in the surveys by using snowball sampling and advertising via different social media platforms and the website of the Medical University of Graz. Based on data obtained from the Austrian Federal Ministry regarding the number of nursing homes in Austria (Federal Ministry for Social Affairs, Health, Care and Consumer Protection 2019), the power calculation performed separately for both surveys led to an ideal sample size of 383 frontline nursing staff from all included settings. The results presented in this paper are based on complete data extracted from the first and the second online surveys from nursing staff who worked in nursing homes.

### Data collection and instruments

The first online survey was conducted between 12 May 2020 and 13 July 2020 and the second online survey was executed between 12 November 2020 and 2 March 2021. We used the software LimeSurvey (LimeSurvey GmbH, Hamburg, Germany) to generate the online surveys.

We collected data on demographic aspects, such as age, sex, work setting (e.g., nursing home), qualification (e.g., registered nurses, nursing aids, nursing students) and job experience measured in years (< 5 years, 5–10 years, 11–20 years, or > 20 years). In addition, questions about whether COVID-19 symptoms were experienced (Yes/No), COVID-19 testing was performed (Yes/No) and whether the nursing staff had cared for suspected/affected COVID-19 cases (Yes/No) were included. Furthermore, we included questions about the personal protective interventions performed, such as the use of masks, and about the general protective interventions performed, such as monitoring the residents’ symptoms. These questions were based on documents published by the WHO (2020a, b) and the Austrian Federal Ministry for Social Affairs, Health, Care and Consumer Protection (2020a, b). The questionnaire can be found in supplement 1.

The stress perceived by nursing staff was measured using the Perceived Stress Scale (PSS), a scale that is available in the German language (Schneider et al., 2017). The PSS consists of 10 items which are rated on a 5-point Likert scale (0 = never; 1 = almost never; 2 = sometimes; 3 = fairly often; 4 = very often) (Klein et al. 2016). Values of 0–13 points indicate a low stress level, 14–26 a moderate stress level, and 27–40 a high perceived stress level. Previous studies with the German PSS reported good internal consistency with a Cronbach’s alpha of 0.84 and a good construct as well as concurrent validity. Furthermore, the scale is practical, as it only includes 10 items (Klein et al. 2016). The Cronbach’s alpha for our sample is 0.88.

### Data analysis

The data cleaning and analysis was performed with the IBM SPSS Statistics 26 (Armonk, New York, USA) software for Windows. First, we conducted a descriptive analysis of all data by carrying out χ^2^-tests and a Mann-Whitney *U* test, due to the nonparametric distribution of the data. Second, both univariate and multivariate logistic regression analyses were performed. All variables that were identified as significant in the descriptive analysis were included in the univariate analysis. Afterwards, the variables identified as significant in the univariate analysis were tested for multicollinearity. Variance inflation factors (VIF) lower than four were defined as indicating non-multicollinearity between the variables (Hair et al. 2016). The stepwise multivariate logistic regression analysis was carried out with the inclusion of only the significant variables identified in the univariate analysis (Field 2005). Odds ratios (OR) with 95% confidence intervals (CI) were calculated and the Hosmer-Lemeshow goodness of fit test was performed to indicate the fit of the final model. *P*-values lower than 0.05 were considered as statistically significant.

### Ethical considerations

The data collection was performed anonymously, and IP addresses were not stored. We asked all participants to provide their written informed consent in order to comply with recognized standards and the Declaration of Helsinki. The study was approved by the ethics board of the Medical University of Graz (32–386 ex 19/20 and 33–118 ex 20/21).

## Results

In total, 749 nursing home staff took part in the surveys: 449 participated in the survey during the first wave and 300 during the second wave. The median age of the participating nursing staff was 41 years and most of the nurses (80% and 83%, respectively) were female with no significant differences noted between the first and second waves (*p* = 0.296). The staff working hours were significantly greater in the second wave (*p* < 0.001). During the first wave, 45.4% of the staff worked more than 40 h/week, while 62.7% worked more than 40 h/week during the second wave. The percentage of nursing staff who experienced COVID-19 symptoms and performed tests and the percentage of persons who cared for residents with COVID-19 were also significantly higher in the second wave (*p* < 0.001) (Table 1).

**Table 1:** Sample characteristics of the participating nursing home staff

	Nursing home staff (*N* = 749)		
	1st wave (*n* = 449)	2nd wave (*n* = 300)	*p*-value
*Median age in years IQR*	41 (33–49)	41 (35–50)	0.246
*Female % (n)*	80.0 (359)	83.0 (249)	0.296
*Qualification % (n)*			
Nurse	65.3 (293)	69.3 (208)	0.026
Nursing aid	29.2 (131)	29.0 (87)	
Nursing student	5.6 (25)	1.7 (5)	
*Job experience % (n)*			
< 5 years	20.3 (91)	9.7 (29)	0.002
5–10 years	18.9 (85)	20.0 (60)	
11–20 years	28.5 (128)	34.0 (102)	
> 20 years	32.3 (145)	36.3 (109)	
*Working hours during the pandemic % (n)*			
˂ 20 h	7.6 (34)	4.6 (13)	< 0.001
21–40 h	47.0 (211)	32.6 (98)	
> 40 h	45.4 (204)	62.7 (188)	
*COVID-19 symptoms % (n)*			
Yes	9.8 (44)	18.7 (56)	< 0.001
*Tested on COVID-19 % (n)*			
Yes	40.8 (183)	94.7 (284)	< 0.001
*Care for COVID-19 residents % (n)*			
Yes	48.8 (219)	75.7 (227)	< 0.001

*IQR* interquartile range, *COVID-19* Coronavirus disease-2019

**Fig. 1:**
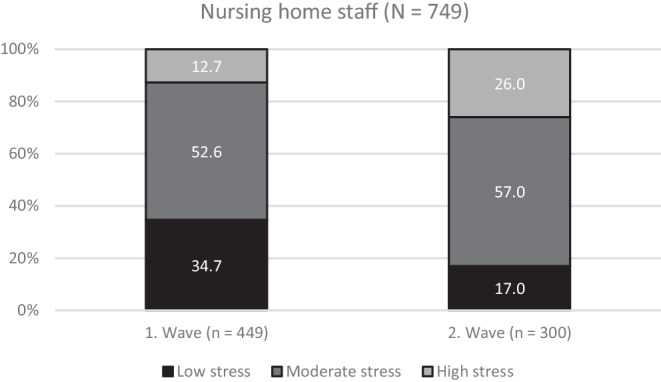
Perceived stress of nursing staff

The perceived stress among nursing staff was significantly higher (*p* < 0.001) in the second wave as compared to the first wave. About one quarter (26.0%) of the respondents reported experiencing a high stress level in the second wave as compared to 12.7% in the first wave (Fig. 1).

During the first wave of COVID-19, the reported high stress level was significantly associated with the female sex (*p* = 0.003) and caring for COVID-19 residents (*p* = 0.041). In the second wave, a significant association between a younger age (*p* = 0.015) and a high stress level was found. Differences in the stress perceived by individual nursing staff who cared for residents with COVID-19 were also observed, but these differences were not significant (*p* = 0.067) (Table 2).

**Table 2:** Comparison of general characteristics between low to moderate and high stress, stratified for wave 1 (spring 2020) and wave 2 (autumn 2020)

	1st wave (*n* = 449)			2nd wave (*n* = 300)		
	Low/moderate stress (*n* = 392)	High stress (*n* = 57)	*p*-value	Low/moderate stress (*n* = 222)	High stress (*n* = 78)	*p*-value
*Median age in years IQR*	41 (33–49)	39 (29–45)	0.063	43 (35–50)	38 (32–48)	0.015
*Female % (n)*	77.8 (305)	94.7 (54)	0.003	82.9 (184)	83.3 (65)	0.927
*Qualification % (n)*						
Nurse	66.1 (259)	59.6 (34)	0.623	69.4 (154)	69.2 (54)	0.392
Nursing aid	28.6 (112)	33.3 (19)		28.4 (63)	30.8 (24)	
Nursing student	5.4 (21)	7.0 (4)		2.3 (5)	–	
*Job experience % (n)*						
< 5 years	19.4 (76)	26.3 (15)	0.488	8.6 (19)	12.8 (10)	0.378
5–10 years	18.9 (74)	19.3 (11)		20.3 (45)	19.2 (15)	
11–20 years	28.3 (111)	29.8 (17)		32.4 (72)	38.5 (30)	
> 20 years	33.4 (131)	24.6 (14)		38.7 (86)	29.5 (23)	
*Working hours during the pandemic % (n)*						
˂ 20 h	7.9 (31)	5.3 (3)	0.787	5.9 (13)	1.3 (1)	0.482
21–40 h	47.4 (186)	43.9 (25)		31.6 (70)	35.9 (28)	
> 40 h	44.6 (175)	50.9 (29)		62.6 (139)	62.8 (49)	
*COVID-19 symptoms % (n)*						
Yes	9.9 (39)	8.8 (5)	0.780	18.0 (40)	20.5 (16)	0.627
*Tested on COVID-19 % (n)*						
Yes	41.3 (162)	36.8 (21)	0.520	95.5 (212)	92.3 (72)	0.281
*Care for COVID-19 residents % (n)*						
Yes	46.9 (184)	61.4 (35)	0.041	73.0 (162)	83.3 (65)	0.067

*IQR* interquartile range, *COVID-19* Coronavirus disease-2019

The logistic regression analysis results show that nursing staff had a 2.195-fold higher relative chance of experiencing a high stress level in the second wave as compared to nursing staff in the first wave (*p* < 0.001). Caring for COVID-19 residents (OR 1.827; *p* = 0.007) and being female (OR 1.992; *p* = 0.018) also significantly increased the relative chance of experiencing a high stress level. Age was negatively associated with stress, meaning that younger nursing staff were at higher relative chance of having high stress level (OR 0.970; *p* = 0.002) (Table 3).

**Table 3:** Univariate and multivariate logistic regression with perceived stress as the outcome variable (*N* = 749)

	Univariate		Multivariate	
	*p*-value	OR (95% CI)	*p*-value	OR (95% CI)
*Sex*^*a*^	0.024	1.901 (1.089–3.321)	0.018	1.992 (1.126–3.524)
*Age*	0.003	0.973 (0.955–0.990)	0.002	0.970 (0.952–0.989)
*Caring for COVID-19 resident*^*b*^	< 0.001	2.213 (1.459–3.357)	0.007	1.827 (1.180–2.827)
*Wave*^*c*^	< 0.001	2.416 (1.654–3.530)	< 0.001	2.195 (1.473–3.270)

Cox and Snell’s R^2^ 0.058; Nagelkerke’s R^2^ 0.096; Hosmer-Lemeshow test χ^2^ 6.237; *df* = 8; *p* = 0.621

*OR* Odds Ratio, *COVID 19* Coronavirus disease-2019

^a^male as reference category

^b^not caring for COVID-19 resident as reference category

^c^first wave as reference category

In general, the most frequently performed personal protective interventions were self-monitoring for symptoms (98.7%; 97.3%), using gloves (97.6%; 91.3%) and surgical face masks (SFM) (96.9%; 61.0%) and keeping a physical distance from patients/residents (96.7%; 94.3%). Several significant differences were identified when examining the results from the surveys conducted in the first and second waves. We observed that the use of FFP (Filtering Face Piece) masks (*p* < 0.001) and protective glasses (*p* < 0.001) increased and that the usage of gloves (*p* < 0.001), SFM (*p* < 0.001) and other interventions (*p* < 0.001) decreased between the first and second waves (Table 4).

Nursing staff most frequently performed the general protective interventions of monitoring for symptoms (98.9%; 96.7%) and psychological conditions (97.8%; 86.0%). The frequency with which most of the general interventions were performed decreased significantly after the first wave. This drop in frequency was especially noted for specific interventions, such as regularly airing the residents’ rooms (81.7%; 70.3%) and organizing meal times with physical distancing (72.4%; 58.7%). However, the frequency of performing certain interventions, such as regularly taking the residents’ temperatures (51.0%; 62.0%, *p* = 0.003), significantly increased (Table 4).

**Table 4:** Interventions performed by the nursing home staff

	Nursing home staff (*N* = 749)		
	1st wave (*n* = 449)	2nd wave (*n* = 300)	*p*-value
*Personal protective interventions % (n)*			
Self-monitoring of symptoms	98.7 (443)	97.3 (292)	0.188
Use of gloves	97.6 (438)	91.3 (274)	< 0.001
Use of SFM	96.9 (435)	61.0 (183)	< 0.001
Maintain distance	96.7 (434)	94.3 (283)	0.123
Protective shell	63.7 (286)	65.3 (196)	0.647
FFP masks	51.7 (232)	96.3 (189)	< 0.001
Other interventions	46.1 (207)	16.0 (48)	< 0.001
Protective eyewear	29.8 (134)	54.3 (163)	< 0.001
*General protective interventions % (n)*			
Monitoring of residents’ symptoms	98.9 (444)	96.7 (290)	0.034
Observe psychological condition of residents	97.8 (439)	86.0 (258)	< 0.001
Report suspected cases to nursing director	90.0 (404)	86.3 (259)	0.125
Isolate suspected cases	86.2 (387)	82.3 (247)	0.151
Air out rooms two times/day	81.7 (367)	70.3 (211)	< 0.001
Informing residents about protective equipment	81.5 (366)	53.0 (159)	< 0.001
Informing residents about COVID-19	80.4 (361)	57.7 (173)	< 0.001
Instruct residents on how to use SFM	74.2 (333)	70.0 (210)	0.211
Organize meal times with distance	72.4 (325)	58.7 (176)	< 0.001
Taking temperature 2 times/day	51.0 (229)	62.0 (186)	0.003
Other interventions	47.2 (212)	15.7 (47)	< 0.001

*SFM* surgical face masks, *FFP* filtering face piece, *COVID 19* Coronavirus disease-2019

## Discussion

Several aims were achieved by carrying out this study. We were able to describe the stress perceived and the interventions performed by nursing staff, as well as to identify factors associated with the perceived stress levels in Austrian nursing homes in the first and the second waves of the COVID-19 pandemic. We found that nursing staff experienced higher stress levels during the second COVID-19 wave. This higher stress level was associated with the female gender, a younger age and caring for COVID-19 suspected/infected cases. Some protective interventions, such as the use of FFP masks, increased from the first to the second wave, while others decreased, such as the practice of airing the residents’ rooms.

Some differences in the general characteristics of the participating nursing staff were identified in our study when we compared survey responses provided in the first as compared to the second COVID-19 waves. The percentage of nursing staff with COVID-19 symptoms, the percentage that was tested and the percentage that cared for residents with COVID-19, as well as the number of working hours, increased significantly. This increase may be explained by the higher infection rates reported in the second wave (AGES 2021) but also by the increase in testing possibilities. Although the staff working hours are officially limited, exceptions are allowed in challenging times. The resulting prolonged or irregular working hours can have many negative consequences on the health and safety of both nursing staff and the residents they care for (Son et al. 2019). Studies have reported problems among nursing staff, such as staff retention, job dissatisfaction, burn-out and stress (Gferer and Gferer 2021).

The stress perceived by the nursing staff increased significantly between the first to the second COVID-19 waves: from 12.7% of nursing staff who experienced a high stress level during the first wave to 26% in the second wave. This increase in perceived stress is also underlined by the results of the logistic regression analysis: it shows that nursing staff in the second wave had a 2.195-fold higher relative chance of experiencing a high stress level as compared to nursing staff in the first wave. One might think that the longer working hours during the second wave would be associated with higher stress, but our study findings did not confirm this. In a former analysis of data collected in the first wave, we identified a positive association between working hours and stress (Hödl et al. 2021a). Another study also found that longer working hours are associated with emotional and mental fatigue, disruptions of normal sleeping and waking hours, depression and various illnesses (Harris et al. 2015). In addition, other studies have shown that the patients’ health and safety decreases as the nurses’ working hours increase (Son et al. 2019). The reason why we did not find such an association in this analysis may be the different setting, because the analysis by Hödl et al. (2021a) included hospitals, home care organizations and nursing homes together.

Among staff included in our study, caring for COVID-19 residents significantly increased the relative chance of experiencing a high stress level. This finding may be explained by the fact that nursing staff members were directly confronted with the consequences of the pandemic, which may have increased their stress. Another possible reason for this finding might be that nursing staff who are directly involved in the care of COVID-19-infected persons need to wear full personal protective equipment (PPE), because studies have shown that wearing PPE can cause physical consequences like headache and pain among frontline healthcare workers (Ong et al. 2020). In the long run, such physical consequences are associated with stress (Tian et al. 2020).

In our study findings, age was negatively associated with stress, meaning that the younger the nursing staff are, the higher their relative chance of experiencing a high stress level. This may be due to the fact that younger nursing staff are less experienced and, thus, may be more anxious. Furthermore, experienced nurses may have already acquired coping strategies that they can use to help them handle challenging situations. This explanation is also supported by the results of another study that were published during the COVID-19 pandemic (Shahrour and Dardas 2020).

Women have generally a higher relative chance of experiencing high stress levels at work, at least in part due to their need to ensure compatibility between their family and their job, which became even more demanding and challenging during the COVID-19 pandemic (Cabarkapa et al. 2020). Especially nursing staff with young children might have experienced difficulty maintaining a work-life balance in the first and second waves of the COVID-19 pandemic, which may have also led to a high stress level. Furthermore, also other stressors like the inability of recreational activities like going out or meeting friends could have influenced the perceived stress level.

In addition, we described the use of personal and general protective interventions. The frequency of use of FFP masks significantly increased in the second wave, while the frequency of use of SFM significantly decreased. This occurred as a result of a national regulation which was issued in February 2021 and indicated that FFP masks need to be worn in closed rooms instead of SFM (Federal Ministry for Social Affairs, Health, Care and Consumer Protection 2020a). The frequency of using other protective interventions decreased, such as the practices of regularly airing the residents’ rooms and organizing meal times with physical distancing. This may be due to changes in protocols and standards in the respective institutions. Furthermore, the season of the second wave (winter) might have led to a decrease in the practice of airing out the residents’ rooms.

This study is one of the first to describe the stress perceived and interventions performed by nursing staff in Austrian nursing homes in the first and second waves of the COVID-19 pandemic. Knowledge about the differences and factors associated with an increased perceived stress level can help to initiate interventions that can help nursing staff to overcome stressful situations and prevent them from resigning from the job. A recently published study from Austria indicated that 64% of nursing staff thought about quitting the job during the COVID-19 pandemic (Gferer and Gferer 2021). Furthermore, we know that stress is associated with the duration of a pandemic. This shows the necessity of initiating stress relief interventions, such as supervision, psychological help, stress relief measurements and financial incentives, in future pandemics as early as possible to prevent extremely high stress levels and the resulting physical consequences (Harris et al. 2015) as well as resignations (Gferer and Gferer 2021). This is extremely important in times of nursing shortages (Catton 2020). For future research, it is recommended to identify effective interventions that can be carried out to address or overcome stressful situations among nursing staff and to recommend their use and application.

Some factors limit our study results. Due to the cross-sectional design of the surveys, it is not possible to describe causal relationships. Furthermore, we could not include qualitative data on the nursing staff’s perceived stress, although this would have complemented the quantitative results of the surveys and potentially deepened the knowledge about the perceived stress. Additionally, we did not have strict inclusion criteria which may have influenced the interpretability. Therefore, in the future, a mixed-method study is recommended in which quantitative and qualitative data collection methods are combined. The samples taken in the first and second waves were not paired and matching was not performed, which may also limit the validity of our findings.

## Conclusion

This study enabled us to describe the stress perceived and interventions performed by nursing staff in Austrian nursing homes in the first and the second waves of the COVID-19 pandemic. Our results show that nursing staff experienced more stress during the second wave, indicating that the duration of a pandemic plays a fundamental role in the coping process and illustrating the heavy burden a long pandemic places on nursing staff. Furthermore, our findings demonstrate that younger age, being female and caring for COVID-19 patients are significantly associated with a higher stress level among nursing staff. The results of this secondary data analysis demonstrate the critical need for nursing management to provide appropriate supportive interventions such as supervision, psychological help, stress relief measures and financial incentives, for nursing staff. This is true in general, but especially for the identified high-risk groups (WHO & International Labour Organization 2021).

## Supplementary Information

Supplement 1: Questionnaire “Nursing Care during the COVID-19 pandemic”. The used questionnaire for both surveys is provided as a supplementary file.

## References

[CR1] AGES Dashboard COVID19 (2021). Datenstand des Epidemiologischen Meldesystems. https://covid19-dashboard.ages.at/. Accessed 12 Dec 2021.

[CR2] Bagchi, S., Mak, J., Li, Q., Sheriff, E., Mungai, E., Anttila, A., Soe, M. M., Edwards, J. R., Benin, A. L., Pollock, D. A., Shulman, E., Ling, S., Moody-Williams, J., Fleisher, L. A., Srinivasan, A., & Bell, J. M. (2021). Rates of COVID-19 Among Residents and Staff Members in Nursing Homes—United States, May 25–November 22, 2020. https://www.cdc.gov/mmwr/volumes/70/wr/mm7002e2.htm. Accessed 12 Dec 2021. 10.15585/mmwr.mm7002e2 PMC7808710 33444301

[CR3] Bauer S, Eglseer D, Hödl M (2020). Pflege während der COVID-19 Pandemie – Eine besondere Herausforderung. ProCare.

[CR5] Cabarkapa S, Nadjidai SE, Murgier J, Ng CH (2020). The psychological impact of VOCID-19 and other viral epidemics on frontline healthcare workers and ways to address it: a rapid systematic review. Brain, Behav Immun Health.

[CR6] Catton H (2020). Global challenges in health and health care for nurses and midwives everywhere. Int Nurs Rev.

[CR7] Centers for Disease Control and Prevention (2021). People with certain medical conditions. https://www.cdc.gov/coronavirus/2019-ncov/need-extra-precautions/people-with-medical-conditions.html. Accessed 13 Dec 2021.

[CR8] Comas-Herrera, A., Zalakaín, J., Lemmon, E., Henderson, D., Litwin, C., Hsu, A. T., Schmidt, A. E., Arling, G., Kruse, F., & Fernández, J. L. (2021). Mortality associated with COVID-19 in care homes: international evidence. https://ltccovid.org/wp-content/uploads/2021/02/LTC_COVID_19_international_report_January-1-February-1-1.pdf. Accessed 5 Dec 2021.

[CR9] Federal Ministry for Social Affairs, Health, Care and Consumer Protection (2019). Pflegepersonal-Bedarfsprognose für Österreich. https://broschuerenservice.sozialministerium.at/Home/Download?publicationId=722. Accessed 5 Dec 2021.

[CR10] Federal Ministry for Social Affairs, Health, Care and Consumer Protection (2020a). Empfehlung zu COVID-19 Schutzmaßnahmen für Pflege und Betreuung. https://www.sozialministerium.at/Informationen-zum-Coronavirus/Coronavirus---Fachinformationen.html. Accessed 5 Dec 2021.

[CR11] Federal Ministry for Social Affairs, Health, Care and Consumer Protection (2020b). Empfehlungen zur schrittweisen Lockerung der aufgrund der COVID-19 Pandemie erlassenen Besuchsbeschränkungen in Alten- und Pflegeheimen ab 4. Mai 2020. http://www.arge-heime-steiermark.at/de/wir/228_502_content_BMSGPK-Empfehlung-Besuchslockerung-fuer-APH.aspx?LNG=. Accessed 5 Dec 2021.

[CR12] Field A (2005). Discovering statistics using SPSS.

[CR13] Galehdar N, Toulabi T, Kamran A, Heydari H (2021). Exploring nurses’ perception of taking care of patients with coronavirus disease (COVID-19): a qualitative study. Nurs Open.

[CR14] Gferer, A., & Gferer, N. (2021). Gesundheits- und Krankenpfleger*innen während der Covid-19 Pandemie in Österreich – Arbeitssituation und Gedanken an einen Ausstieg aus dem Pflegeberuf. *Österreichische Pflegezeitschrift*, *04*, 18–24. 10.1007/s00735-021-1378-6 PMC8445791 34548761

[CR15] Hair JF, Hult GTM, Ringle C, Sarstedt M (2016). A primer on partial least squares structural equation modeling (PLS-SEM).

[CR16] Harris R, Sims S, Parr J, Davies N (2015). Impact of 12h shift patterns in nursing: a scoping review. Int J Nurs Stud.

[CR17] Hödl M, Eglseer D, Bauer S (2021). Associations between personal protective equipment and nursing staff stress during the COVID-19 pandemic. J Nurs Manag.

[CR18] Hödl M, Bauer S, Eglseer D, Fangmeyer M, Flatscher-Thöni M, Kellerer J, Kreyer C, Müller G, Pallauf M, Rohringer M, Toromanova A, Schoberer D (2021). Make nursing practice visible through nursing science in times of COVID-19. Wien Med Wochenschr.

[CR19] Hödl M, Bauer S, Eglseer D (2021). Influence of nursing staff working hours on stress levels during the COVID-19 pandemic: a cross-sectional online survey. HeilberufeScience.

[CR20] Klein EM, Brahler E, Dreier M, Reinecke L, Muller KW, Schmutzer G, Wölfing K, Beutel ME (2016). The German version of the Perceived Stress Scale—psychometric characteristics in a representative German community sample. BMC Psychiatry.

[CR21] Leskovic L, Erjavec K, Leskovar R, Vukovič G (2020). Burnout and job satisfaction of healthcare workers in Slovenian nursing homes in rural areas during the COVID-19 pandemic. Ann Agric Environ Med.

[CR22] McGilton K, Escrig-Pinol A, Gordon A, Chu C, Zuniga F, Sanchez M, Boscart V, Meyer J, Corazzini K, Jacinto A, Spilsbury K, Backman A, Scales K, Fagertun A, Wu B, Edvardsson D, Lepore M, Leung A, Siegel E, Noguchi-Watanabe M, Wang J, Bowers B (2020). Uncovering the devaluation of nursing home staff during COVID-19: are we fuelling the next health care crisis?. J Am Med Dir Assoc.

[CR4] News, B. B. C. (2020). Coronavirus: Carers ‘overwhelmed’ at response to caravan appeal. https://www.bbc.com/news/uk-england-hereford-worcester-52425119. Accessed 12 Nov 2021.

[CR23] Ong J, Bharatendu C, Goh Y, Tang JY, Sooi K, Tan Y, Tan BYQ, Teoh H-L, Ong ST, Allen DM, Sharma V (2020). Headaches associated with personal protective equipment—A cross-sectional study among frontline healthcare workers during COVID-19. Headache.

[CR24] Ouslander JG, Grabowski DC (2020). COVID-19 in nursing homes: calming the perfect storm. J Am Geriatr Soc.

[CR25] Rimmer, M., & Madden, S. (2021). Coronavirus: The NHS staff living away from homes and families. https://www.bbc.com/news/uk-england-52280264. Accessed 5 Dec 2021.

[CR99] Schneider EE, Schönfelder S, Wolf M, Wessa M (2017). All stressed out? Introducing a German version of the perceived stress scale: Validation, psychometric properties and sample differences in healthy and clinical populations. Psychoneuroendocrinology.

[CR26] Schoberer D, Reiter L, Thonhofer N, Hödl M (2022). Occupational relationships and working duties of nursing management staff during the COVID-19 pandemic: a qualitative analysis of survey responses. J Adv Nurs.

[CR27] Shahrour G, Dardas LA (2020). Acute stress disorder, coping self-efficacy and subsequent psychological distress among nurses among COVID-19. J Nurs Manag.

[CR28] Son Y-J, Lee EK, Ko Y (2019). Association of working hours and patient safety competencies with adverse nurse outcomes: a cross-sectional study. Int J Environ Res Public Health.

[CR29] Tian Z, Kim B-Y, Bae M-J (2020). A study on the effect of wearing masks on stress response. Int J Eng Res Techn.

[CR30] WHO & International Labour Organization (2021). COVID-19: occupational health and safety for health workers Interim guidance. https://www.who.int/publications/i/item/WHO-2019-nCoV-HCW_advice-2021.1. Accessed 5 Nov 2021.

[CR33] WHO (2021). WHO Coronavirus (COVID-19) Dashboard. https://covid19.who.int/. Accessed 5 Dec 2021.

[CR31] World Health Organization (2020a). Home care for patients with COVID-19 presenting with mild symptoms and management of their contacts. https://www.who.int/publications/i/item/home-care-for-patients-with-suspected-novel-coronavirus-(ncov)-infection-presenting-with-mild-symptoms-and-management-of-contacts. Accessed 23 Nov 2021.

[CR32] World Health Organization (2020b). Preparedness, prevention and control of coronavirus disease (COVID-19) for refugees and migrants in non-camp settings. https://www.who.int/publications/i/item/preparedness-prevention-and-control-of-coronavirus-disease-(covid-19)-for-refugees-and-migrants-in-non-camp-settings. Accessed 5 Nov 2021.

