# Supplementary material for: Stress among nursing staff and interventions in Austrian nursing homes: Results of a survey in the first and the second waves of COVID-19
Source: HeilberufeScience. 2023 Feb 2;14(1-2):47–55. doi: 10.1007/s16024-022-00395-x (PMC9893177; doi:10.1007/s16024-022-00395-x)
Supplement: Supplementary file 1 — Supplement 1: Questionnaire “Nursing Care during the COVID-19 pandemic”. The used questionnaire for both surveys is provided as a supplementary file. [file 16024_2022_395_MOESM1_ESM.docx]

**Supplement 1: FRAGEBOGEN „Pflegerische Situation während der COVID-19-Pandemie“**

1. Möchten Sie an dieser Onlinebefragung teilnehmen?
2. In welchem Bundesland arbeiten Sie?
3. In welcher Art von Gesundheitseinrichtung sind Sie beschäftigt?
4. Zu welcher Berufsgruppe gehören Sie?
5. Wie lange sind Sie bereits in einem der oben genannten Gesundheitsberufe bereits tätig?
6. Wie viele Stunden sind Sie pro Woche in der pflegerischen Praxis beschäftigt?
7. Wie viele Stunden haben Sie seit der Corona-Pandemie in Österreich (ca. Mitte März 2020) durchschnittlich gearbeitet?
8. In welchem Jahr sind Sie geboren?
9. Bitte geben Sie Ihr Geschlecht an.
10. Gibt es in Ihrer Einrichtung einen Leitfaden zur Identifizierung und Behandlung von Verdachtsfällen bzw. COVID-19 Betroffenen?
11. Gab oder gibt es in Ihrer Einrichtung eine Schulung zu generellen Schutzmaßnahmen (Hygienevorschriften, Besuchsregeln etc.) bezüglich COVID-19?
12. Gab es zu Beginn (Mitte März 2020) der COVID-19 Pandemie folgende Schutzausrüstung:

| Einmalkittel/Schutzmantel |
| --- |
| Handschuhe |
| Mund-Nasen-Schutz |
| ….. |

1. Gibt es zum derzeitigen Zeitpunkt der COVID-19 Pandemie folgende Schutzausrüstung:

| Einmalkittel/Schutzmantel |
| --- |
| Handschuhe |
| Mund-Nasen-Schutz |
| …. |

1. Wie lange tragen Sie im Durchschnitt einen Mund-Nasen-Schutz, bevor Sie einen Neuen nutzen?
2. Wie lange tragen Sie im Durchschnitt eine FFP-Maske, bevor Sie eine Neue nutzen?
3. Gab oder gibt es in Ihrer Einrichtung eine Schulung zum Umgang mit Schutzausrüstungen (Arten/Zweck der Schutzausrüstung, Anziehen, Ausziehen, Entsorgen)?
4. Wurde aufgrund von COVID-19 die Dauer der jeweiligen Dienstzeiten verändert?
5. Welche Begleitmaßnahmen zum Schutz vor psychischer und physischer Überlastung der Mitarbeiter*innen wurden in Ihrer Einrichtung aufgrund von COVID-19 gesetzt?

- Transparenz über die Maßnahmen
- Zeitgerechte Informationsweitergabe seitens des Managements
- ….

1. Waren Sie jemals an der pflegerischen Versorgung eines Verdachtsfalls/COVID-19 Betroffenen beteiligt?
2. Welche organisatorischen Maßnahmen haben Sie in Bezug auf COVID-19 durchgeführt?

- An- und Zugehörige über Regelungen und Maßnahmen informiert
- Zutritts- und Besuchsverbote an den Eingängen an den Eingängen ausgehängt
- …..

1. Welche persönlichen Schutzmaßnahmen haben Sie in Bezug auf COVID-19 durchgeführt?

- Selbstbeobachtung bezüglich Symptomen (z.B. Fieber)
- Abstandsregeln eingehalten
- …..

1. Welche allgemeinen Schutzmaßnahmen haben Sie in Bezug auf COVID-19 durchgeführt?

- Händehygiene nach dem Betreten der Räumlichkeiten der Patient*in/Bewohner*in
- Händehygiene vor und nach dem Patient*innen/Bewohner*innenkontakt
- ….

1. Hatten Sie jemals Symptome der COVID-19 Erkrankung?
2. Wurden Sie positiv auf COVID-19 getestet?
3. Die folgenden Fragen beschäftigen sich mit Ihren Gedanken und Gefühlen während der COVID-19 Pandemie. Bitte geben Sie für jede Frage an, wie oft Sie in entsprechender Art und Weise gedacht oder gefühlt haben.

|  | **Nie** | **Fast nie** | **Manchmal** | **Ziemlich oft** | **Sehr oft** |
| --- | --- | --- | --- | --- | --- |
| Wie oft waren Sie im letzten Monat aufgewühlt, weil etwas unerwartet passiert ist? |  |  |  |  |  |
| Wie oft hatten Sie im letzten Monat das Gefühl, nicht in der Lage zu sein, die wichtigen Dinge in Ihrem Leben kontrollieren zu können? |  |  |  |  |  |
| ….. |  |  |  |  |  |
